# Supplementary material for: Chimpanzees produce diverse vocal sequences with ordered and recombinatorial properties
Source: Commun Biol. 2022 May 16;5:410. doi: 10.1038/s42003-022-03350-8 (PMC9110424; doi:10.1038/s42003-022-03350-8)
Supplement: Supplementary file 2 — Supplementary Information [file 42003_2022_3350_MOESM2_ESM.pdf]

SUPPLEMENTARY MATERIAL for:

Chimpanzees produce diverse vocal sequences with ordered and recombinatorial properties

Cédric Girard-Buttoz[1,2,3,4&], Emiliano Zaccarella[5&], Tatiana Bortolato[2,3,4], Angela D. Friederici[5]
Roman M. Wittig[2,3,4], Catherine Crockford[1, 2, 3,4]

Supplementary discussion

Two-unit and longer sequences appear throughout the chimpanzee vocal repertoire and are not limited to the well-described four-unit pant-hoot sequence of the chimpanzees includes single hoos (HO) – panted hoos (PH) – climax phase (panted screams (PS) or panted bark (PB)), and let down phase (panted roars (PR) or pants (PN))[43,105]. Whilst our trigram analysis includes three sets of bigrams that can be emitted independently or produced as part of the pant hoot sequence (head: HO_PH, PH_PS; tail: PH_PS), the other bigram GR-PG which appears in tail and head position is not part of pant-hoot sequence. Likewise, many other frequent (above chance) bigrams and trigrams we have identified are never emitted as part of a pant hoot sequence (e.g., HO_PG, GR_PG_PN, GR_PG_PB, PG_PB_BK **Fig. 3A, S3 D, E and F, and S5**).

Supplementary Figures

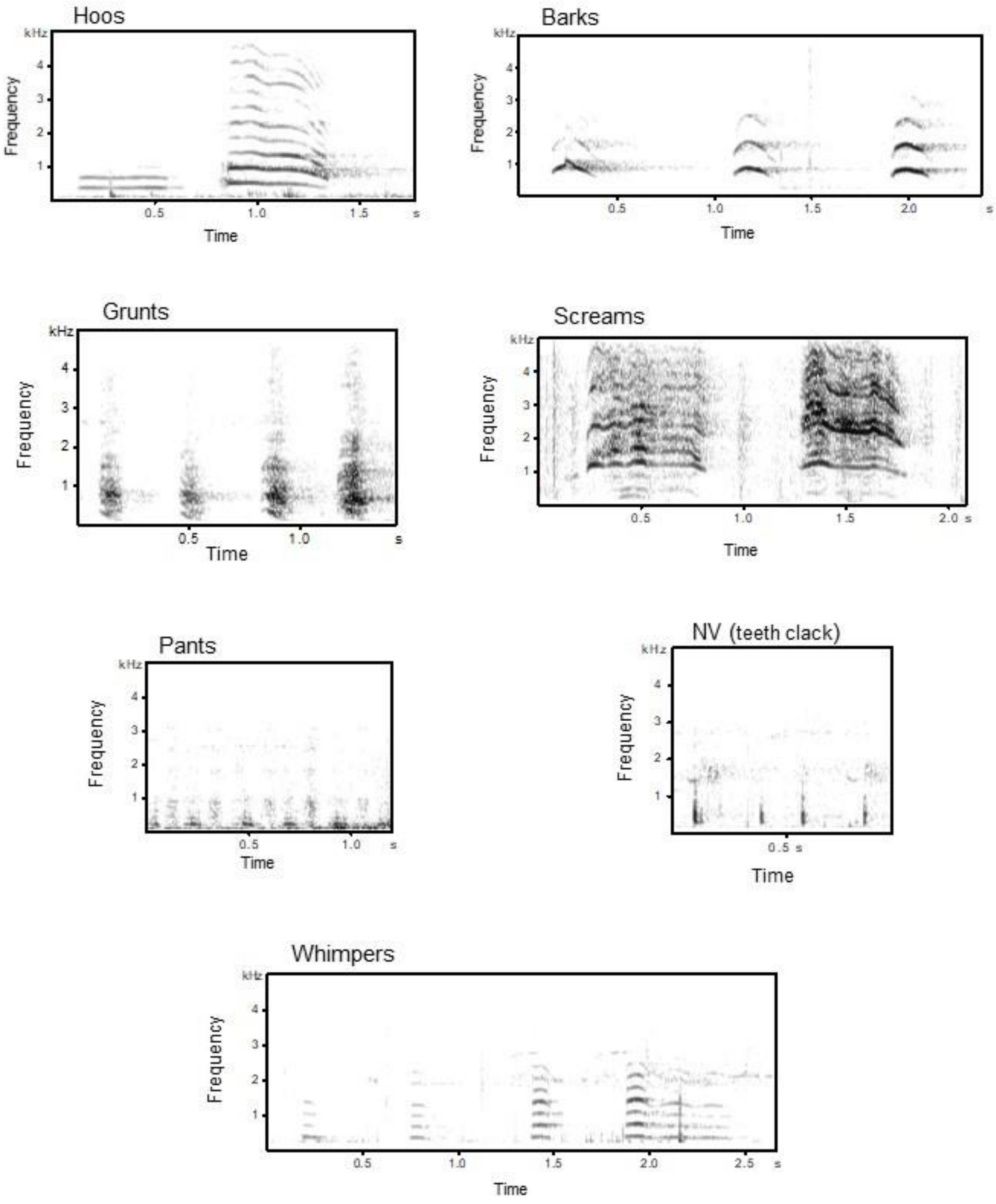

Supplementary Figure 1: Spectrograms of chimpanzee single-unit vocalizations.

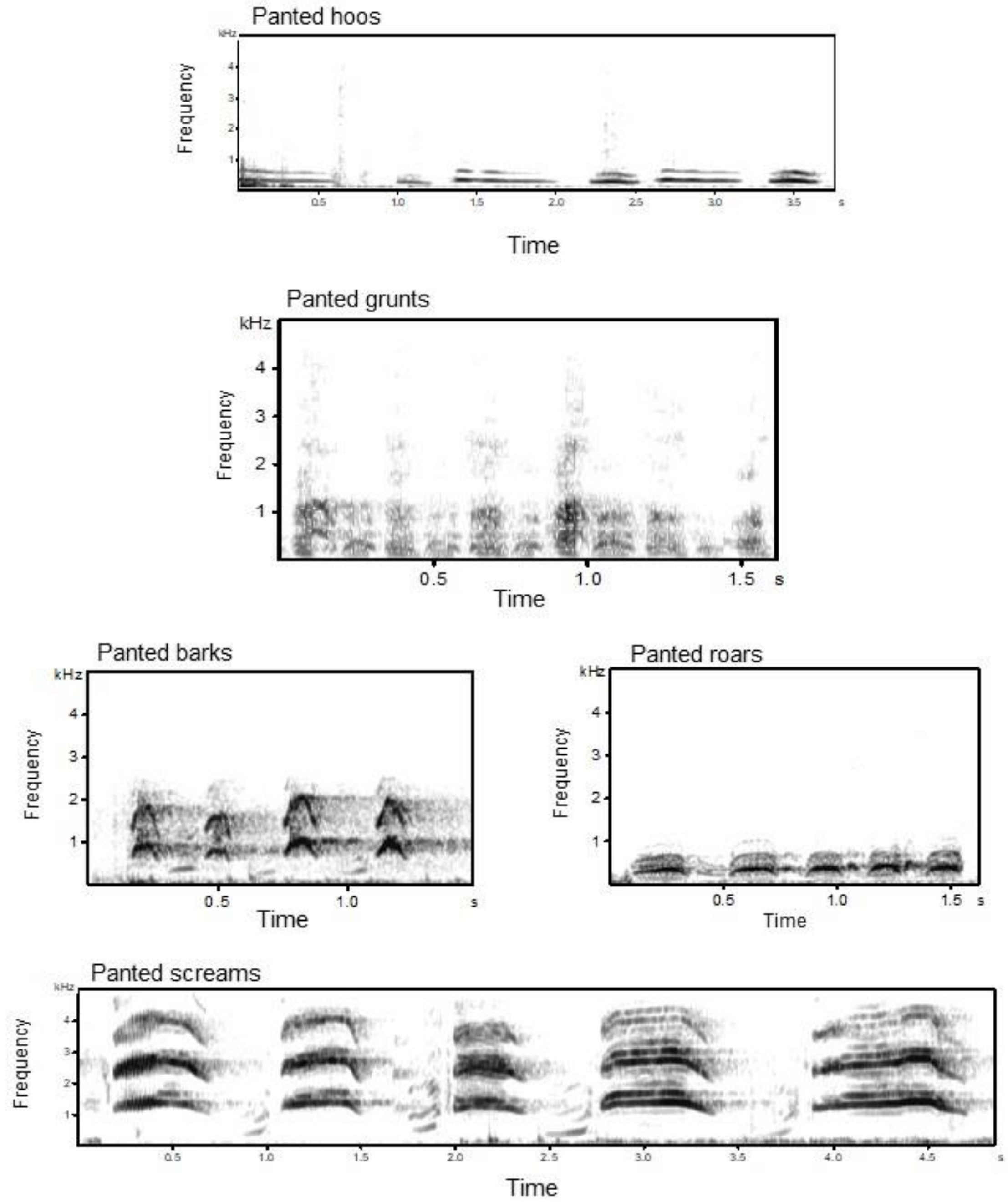

Supplementary Figure 2: Spectrograms of chimpanzee panted-unit vocalizations. Roars only occurred as a panted form.

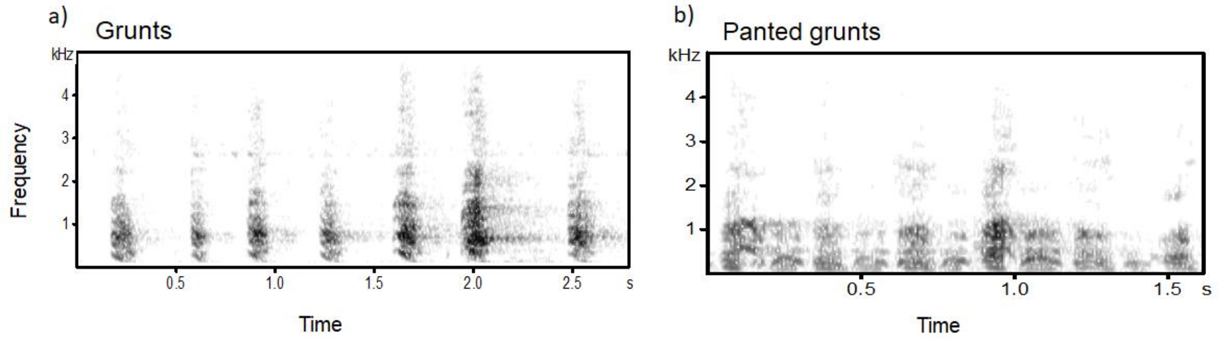

Supplementary Figure 3: An example showing the construction of chimpanzee single units. Spectrograms show (a) a single non-panted call (e.g., series of Grunts) and (b) a single panted call (e.g., series of Panted grunts).

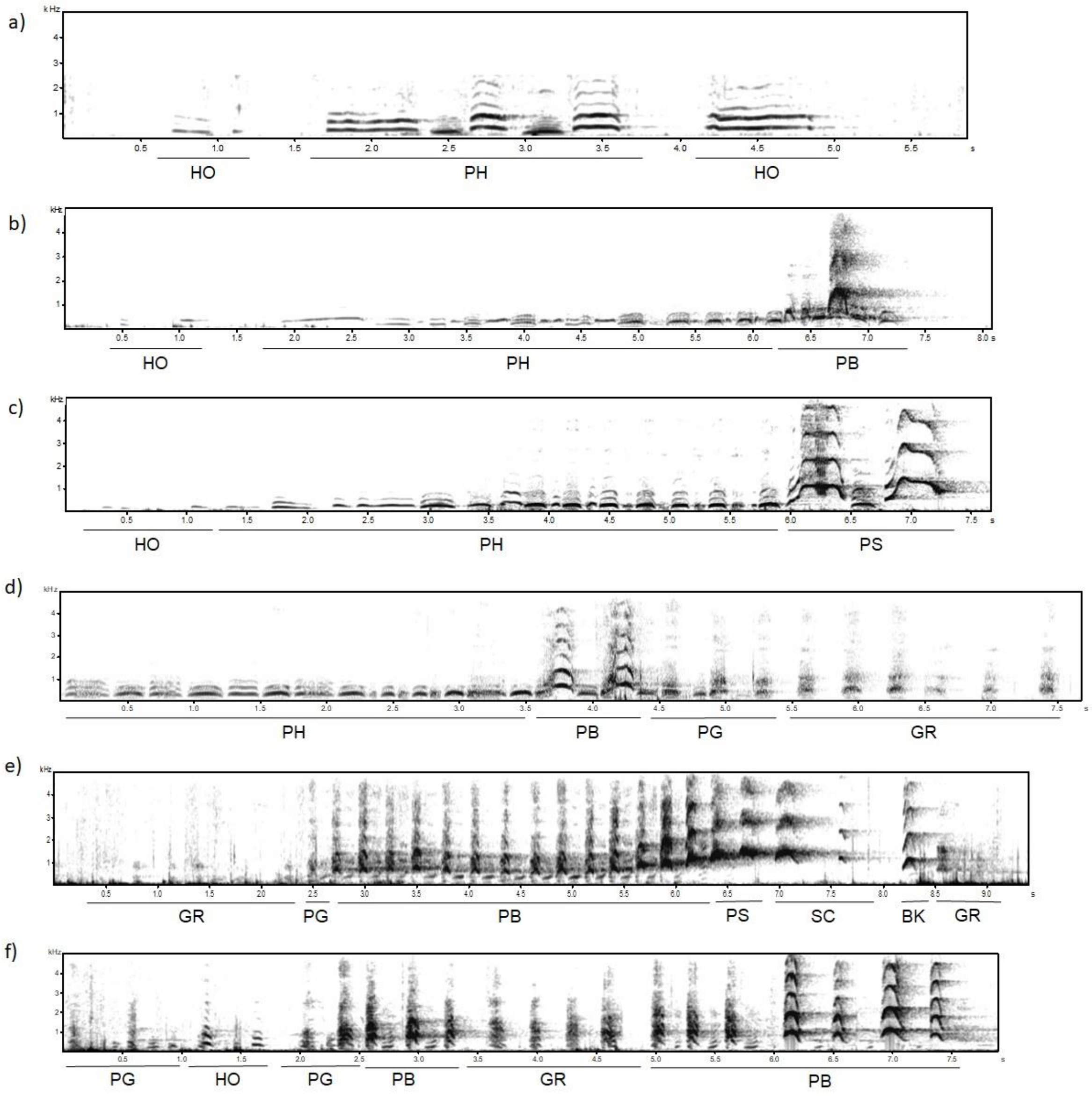

Supplementary Figure 4: An example showing the construction of chimpanzee sequences. Frequency (kHz) in y-axis and time (seconds) in x-axis. Spectrograms (a), (b) and (c) show three-unit sequences (trigrams) composed by Hoos (single hoo in (a) or series or hoos in (b) and (c)) and series of Panted hoos followed by either a Hoo (a), series of Panted barks (b) or series of Panted screams (c). Spectrogram (d) shows a four-unit sequence composed by series of: Panted hoos, Panted barks, Panted grunts and finally Grunts. Spectrograms (e) and (f) show different long sequences without the structure of the classic Pant-

hoot call. The sequence in (e) is composed of Grunts, a single Panted Grunt, Panted barks, Panted screams, Screams, a single Bark and Grunts. Sequence (f) is composed of Panted grunts, Hoos, Panted grunts, Panted barks, Grunts, and Panted barks.

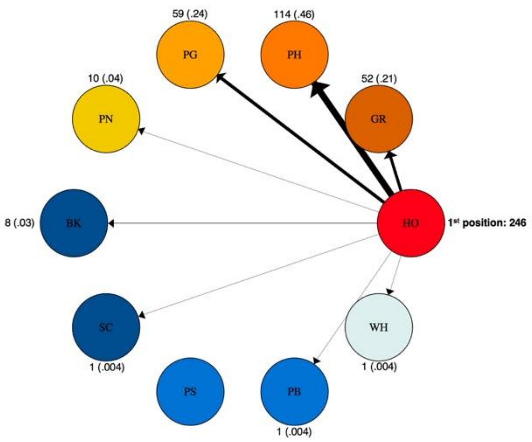

Supplementary Figure 5. Example of two-unit sequences (bigrams) given a certain call in position one (HO). Colours represent the number of times a certain unit is found in the bigram sample (red-to-turquoise). The size of the directional edges (arrows) express the number of times that specific bigram is found in the study (thick-to-thin). The values on each calls express the number of times that the unit is found in position two with HO in position one, with corresponding percent value.

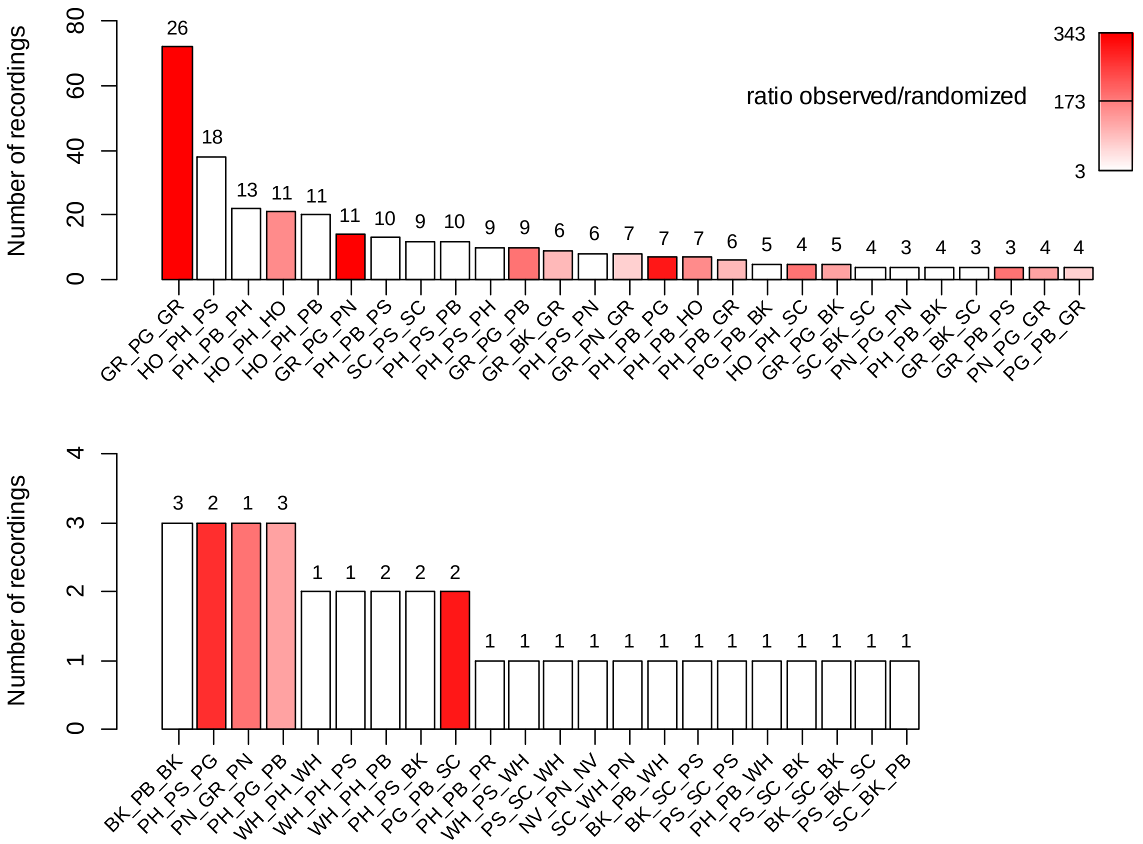

Supplementary Figure 6: Frequency of production of three-unit sequences (trigrams) that are produced above chance (i.e., 95% more likely than by random juxtaposition of single units). The height of each bar corresponds to the number of time each trigram was recorded. The colour gradient in the bars depicts the number of time each trigram was observed divided by the number of time each trigram was present on average in each randomization (averaged over 1000 randomizations). The colour range from the lowest in white (i.e., the trigram was present in the randomization three times less than the number of times it was observed) to the highest ratio in red (i.e., the trigram was present in the randomization 343 times less than the frequency at which it was observed). The number on top of each bar indicates the number of individual that produced each trigram. The abbreviation for the call names (or single units) are as in **Table 1**.

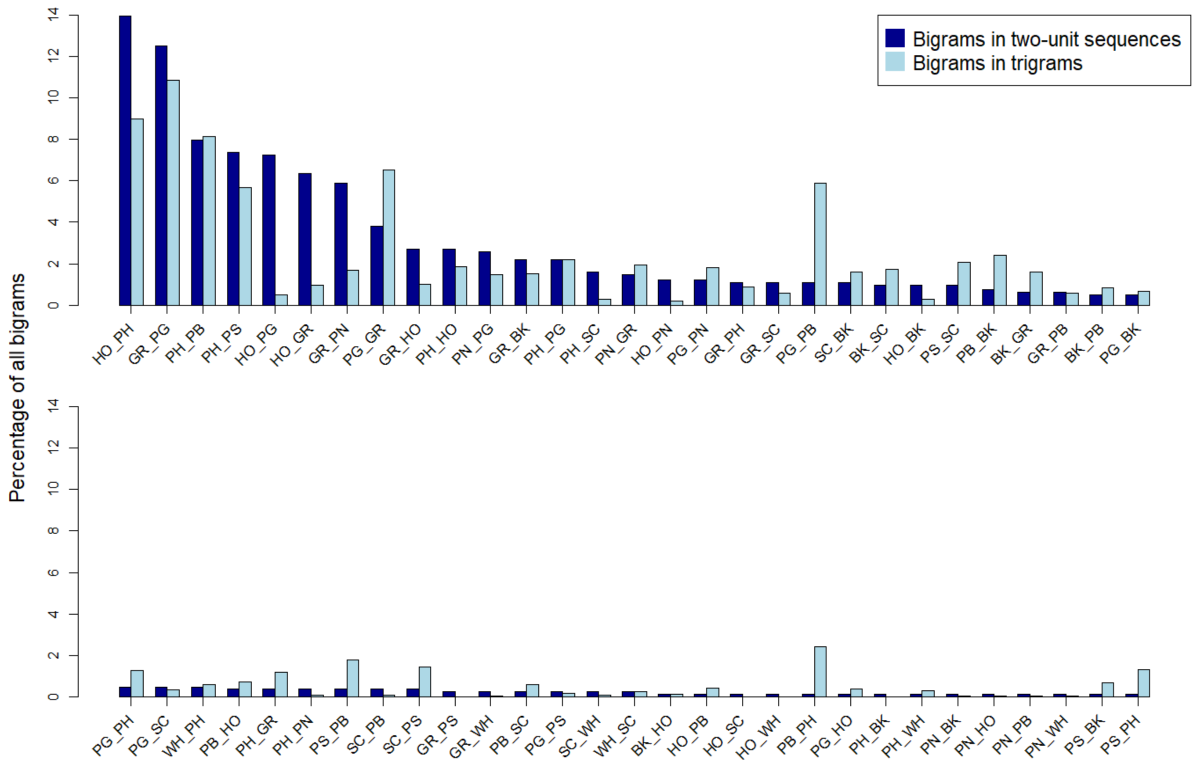

Supplementary Figure 7: Frequency distribution of bigrams in two-unit sequences (dark blue) and of bigrams within three-unit sequences (trigrams, light blue). The height of the bars indicates the percentage of occurrence of each bigram within each frequency distribution as a percentage of all bigrams recorded. The abbreviation for the unit names are as in Table 1.

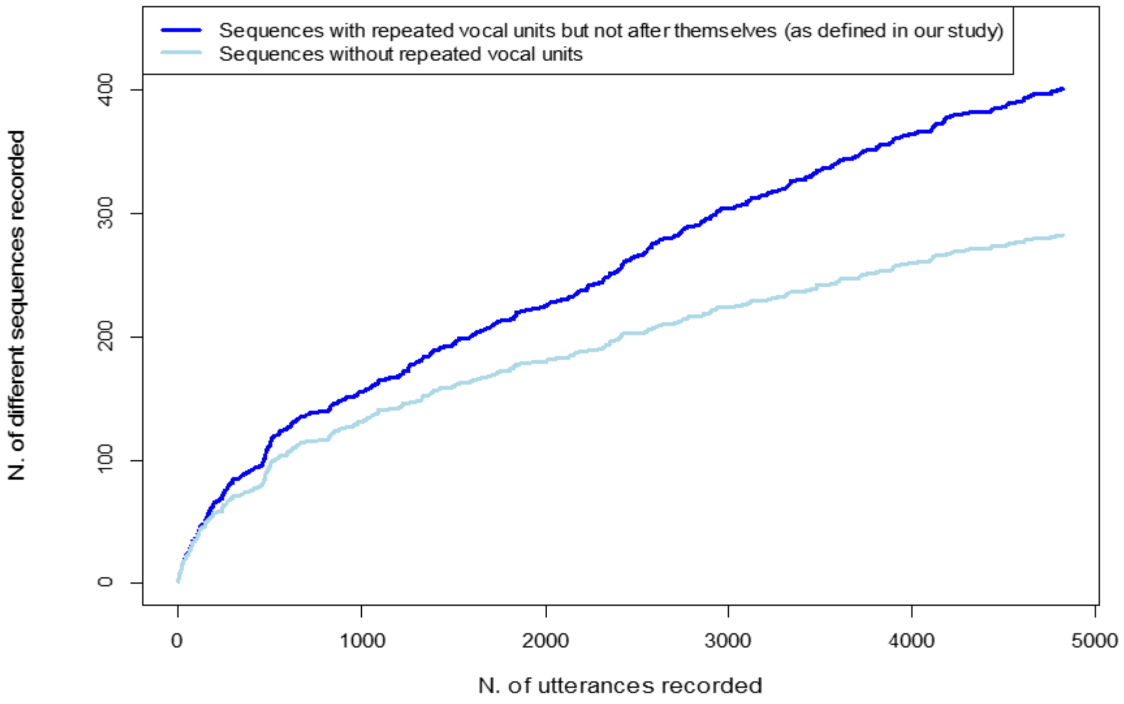

Supplementary Figure 8: Number of different vocal sequences found in the Taï chimpanzee vocal repertoire as a function of the number of utterances recorded.

The dark blue line depicts the number of unique sequences as defined in our study. The sequence considered are the ones where the same single unit can occur more than ones within the sequence but not after itself. For instance, A_A_A_B_C would be coded as A_B_C and be no different to an A_B_C sequence. In contrast, A_B_C_A would be different from A_B_C or from B_C_A since A appears twice in the sequence but not after itself.

The light blue line depicts the number of unique sequences in which the same single unit is not repeated at all. For instance, the sequence A_B_A_C would be considered the same as the sequence A_B_C since the repetition of A in the first sequence would not be taken into account).

We depict here these two quantification methods since both methods are used in the primate vocal communication literature to assess the diversity of sequences produced by other primate species.

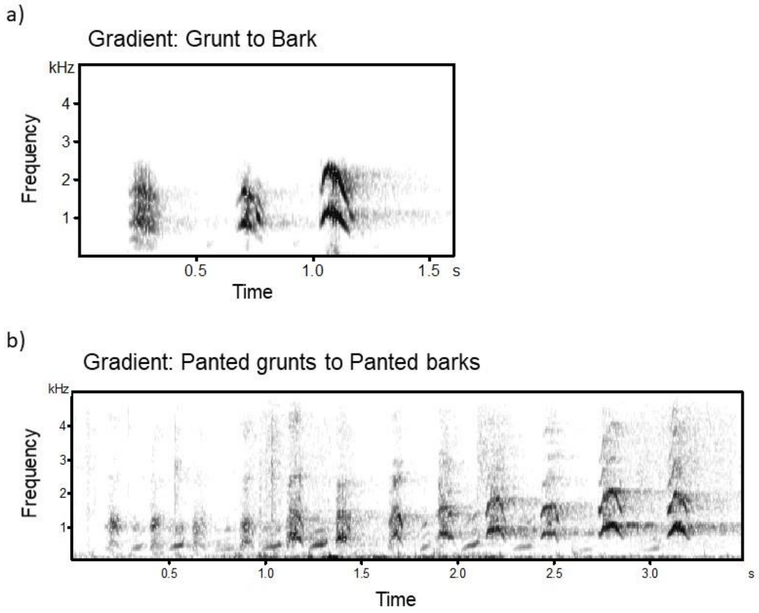

Supplementary Figure 9: Spectrograms of chimpanzees´ graded vocal system showing (a) a gradient from a Grunt to a Bark; and (b) a gradient from Panted-grunts to Panted-barks.

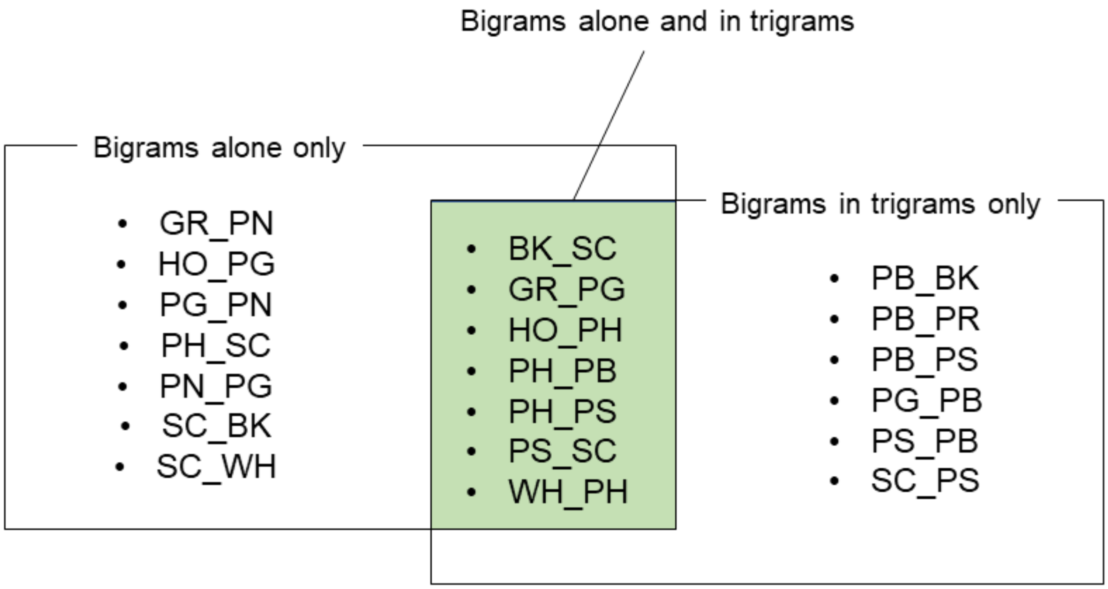

Supplementary Figure 10: Illustration of bigrams produced above chance which were produced only as bigrams alone, as bigrams within trigrams or both.

Supplementary Tables

Supplementary Table 1: Positional occurrences in two-unit sequences (bigrams)

second position (columns); first position (rows)

unit	BK	GR	HO	NV	PB	PG	PH	PN	PR	PS	SC	WH	total
BK		5	1	0	4	0	0	0	0	0	8	0	18
GR	18		22	0	5	102	9	48	0	2	9	2	217
HO	8	52		0	1	59	114	10	0	0	1	1	246
NV	0	0	0		0	0	0	0	0	0	0	0	0
PB	6	0	3	0		0	1	0	0	0	2	0	12
PG	4	31	1	0	9		4	10	0	2	4	0	65
PH	1	3	22	0	65	18		3	0	60	13	1	186
PN	1	12	1	0	1	21	0		0	0	0	1	37
PR	0	0	0	0	0	0	0	0		0	0	0	0
PS	1	0	0	0	3	0	1	0	0		8	0	13
SC	9	0	0	0	3	0	0	0	0	3		2	17
WH	0	0	0	0	0	0	4	0	0	0	2		6
total	48	103	50	0	91	200	133	71	0	67	47	7	817

Supplementary Table 2: Positional occurrences in three-unit sequences (trigrams)

two-unit	Head	Tail	total
HO_PH	94	0	94
GR_PG	105	8	113
PH_PB	62	25	87
PH_PS	36	40	76
PN_PG	10	3	13

Supplementary Table 3: Transitional relationships (forward) in three-unit sequences (trigrams)

two-unit utterance (columns); following_call (rows)

unit	GR_PG	GR_PN	HO_PG	HO_PH	PH_PB	PH_PS	PN_PG
BK	5	0	0	0	4	2	0
GR	72	8	1	6	6	0	4
HO	0	0	2	21	7	0	0
NV	0	1	0	0	0	0	0
PB	10	0	0	20	0	12	0
PG	0	2	0	3	7	3	0
PH	2	0	2	0	22	10	2
PN	14	0	1	0	0	8	4
PR	0	0	0	0	1	0	0
PS	1	0	0	38	13	0	0
SC	1	0	0	5	1	1	0
WH	0	0	0	1	1	0	0
total	105	11	6	94	62	36	10

Supplementary Table 4: Transitional relationships (backward) in three-unit sequences (trigrams)

two-unit utterance

preceding unit

two-unit	GR_PG	GR_PN	HO_PG	HO_PH	PH_PB	PH_PS	PN_PG
BK	0	0	0	0	1	0	0
GR	0	0	0	0	1	0	2
HO	3	2	0	0	20	38	1
NV	0	0	0	0	0	0	0
PB	0	0	0	0	0	0	0
PG	2	0	0	0	1	0	0
PH	1	1	0	0	0	0	0
PN	2	3	0	0	0	0	0
PR	0	0	0	0	0	0	0
PS	0	0	0	0	0	0	0
SC	0	0	0	0	0	0	0
WH	0	0	0	0	2	2	0
total	8	6	0	0	25	40	3
