# Supplementary material for: Chimpanzees produce diverse vocal sequences with ordered and recombinatorial properties
Source: Commun Biol. 2022 May 16;5:410. doi: 10.1038/s42003-022-03350-8 (PMC9110424; doi:10.1038/s42003-022-03350-8)
Supplement: Supplementary file 3 — Description of Additional Supplementary Files [file 42003_2022_3350_MOESM3_ESM.pdf]

Description of Additional Supplementary Files

File name: Supplementary Data 1
Description: Details of single unit and the sequences of different length recorded.

File name: Supplementary Data 2
Description: Source data for figures.
