# Supplementary material for: Chimpanzees produce diverse vocal sequences with ordered and recombinatorial properties
Source: Commun Biol. 2022 May 16;5:410. doi: 10.1038/s42003-022-03350-8 (PMC9110424; doi:10.1038/s42003-022-03350-8)
Supplement: Supplementary file 4 — Supplementary Data 1 [file 42003_2022_3350_MOESM4_ESM.docx]

**Supplementary data S1: Details of single unit and the sequences of different length recorded.**

| **Number of single units in the utterance** | **Number of recordings** | **Number of unique utterance type** | **List of the unique utterance** |
| --- | --- | --- | --- |
| 1 | 3242 | 11 | BK, GR, HO, NV, PB, PG, PH, PN, PS, SC, WH |
| 2 | 817 | 58 | BK_GR, BK_HO, BK_PB, BK_SC, GR_BK, GR_HO, GR_PB, GR_PG, GR_PH, GR_PN, GR_PS, GR_SC, GR_WH, HO_BK, HO_GR, HO_PB, HO_PG, HO_PH, HO_PN, HO_SC, HO_WH, PB_BK, PB_HO, PB_PH, PB_SC, PG_BK, PG_GR, PG_HO, PG_PB, PG_PH, PG_PN, PG_PS, PG_SC, PH_BK, PH_GR, PH_HO, PH_PB, PH_PG, PH_PN, PH_PS, PH_SC, PH_WH, PN_BK, PN_GR, PN_HO, PN_PB, PN_PG, PN_WH, PS_BK, PS_PB, PS_PH, PS_SC, SC_BK, SC_PB, SC_PS, SC_WH, WH_PH, WH_SC |
| 3 | 458 | 104 | BK_PB_BK, BK_PB_WH, BK_PH_PB, BK_SC_BK, BK_SC_PS, GR_BK_GR, GR_BK_PB, GR_BK_SC, GR_HO_GR, GR_PB_PS, GR_PB_SC, GR_PG_BK, GR_PG_GR, GR_PG_PB, GR_PG_PH, GR_PG_PN, GR_PG_PS, GR_PG_SC, GR_PH_GR, GR_PH_HO, GR_PH_PB, GR_PN_GR, GR_PN_NV, GR_PN_PG, GR_SC_GR, HO_BK_HO, HO_BK_PS, HO_BK_SC, HO_GR_HO, HO_GR_PG, HO_GR_PN, HO_PB_BK, HO_PB_PG, HO_PB_PS, HO_PG_GR, HO_PG_HO, HO_PG_PH, HO_PG_PN, HO_PH_GR, HO_PH_HO, HO_PH_PB, HO_PH_PG, HO_PH_PS, HO_PH_SC, HO_PH_WH, HO_PN_GR, HO_PN_PG, NV_PN_NV, PG_BK_GR, PG_BK_SC, PG_GR_PG, PG_PB_BK, PG_PB_GR, PG_PB_PG, PG_PB_SC, PG_PH_GR, PG_PH_PB, PH_GR_PG, PH_GR_PN, PH_PB_BK, PH_PB_GR, PH_PB_HO, PH_PB_PG, PH_PB_PH, PH_PB_PR, PH_PB_PS, PH_PB_SC, PH_PB_WH, PH_PG_BK, PH_PG_GR, PH_PG_HO, PH_PG_PB, PH_PG_PH, PH_PG_PS, PH_PG_SC, PH_PS_BK, PH_PS_PB, PH_PS_PG, PH_PS_PH, PH_PS_PN, PH_PS_SC, PH_SC_GR, PN_GR_BK, PN_GR_PG, PN_GR_PN, PN_GR_SC, PN_PG_GR, PN_PG_PH, PN_PG_PN, PN_PH_PN, PS_BK_SC, PS_SC_BK, PS_SC_PS, PS_SC_WH, SC_BK_PB, SC_BK_SC, SC_PS_GR, SC_PS_SC, SC_WH_PN, WH_PH_HO, WH_PH_PB, WH_PH_PS, WH_PH_WH, WH_PS_WH |
| 4 | 170 | 106 | BK_PN_PB_PH, BK_PS_BK_PN, BK_PS_PB_PG, BK_SC_BK_GR, BK_SC_BK_PB, BK_SC_PS_BK, GR_BK_SC_PS, GR_HO_PH_GR, GR_PB_BK_GR, GR_PB_HO_BK, GR_PB_PS_BK, GR_PG_BK_GR, GR_PG_BK_SC, GR_PG_GR_BK, GR_PG_GR_HO, GR_PG_GR_PG, GR_PG_GR_SC, GR_PG_PB_BK, GR_PG_PB_GR, GR_PG_PB_PG, GR_PG_PB_SC, GR_PG_PH_GR, GR_PG_PH_PB, GR_PG_PH_PG, GR_PG_PN_GR, GR_PG_PS_SC, GR_PG_SC_BK, GR_PH_HO_GR, GR_PH_PG_GR, GR_PN_GR_PG, GR_PN_PG_GR, GR_SC_BK_GR, HO_PB_BK_GR, HO_PB_PS_SC, HO_PH_HO_GR, HO_PH_HO_PG, HO_PH_HO_PN, HO_PH_PB_BK, HO_PH_PB_GR, HO_PH_PB_HO, HO_PH_PB_PG, HO_PH_PB_PH, HO_PH_PB_PS, HO_PH_PG_GR, HO_PH_PG_PB, HO_PH_PG_PN, HO_PH_PS_GR, HO_PH_PS_PB, HO_PH_PS_PG, HO_PH_PS_PH, HO_PH_PS_PN, HO_PH_PS_SC, HO_PH_WH_SC, HO_PS_BK_GR, HO_PS_PB_PS, PB_GR_PG_BK, PG_GR_PG_PB, PG_GR_PG_PN, PG_GR_PN_SC, PG_PB_BK_GR, PG_PB_PG_GR, PG_PB_PS_GR, PG_PB_PS_SC, PG_PH_GR_HO, PG_PH_PB_BK, PG_SC_BK_SC, PH_HO_PH_HO, PH_PB_HO_PG, PH_PB_PG_HO, PH_PB_PG_PN, PH_PB_PH_GR, PH_PB_PH_PG, PH_PB_PH_PS, PH_PB_PN_PS, PH_PB_PS_HO, PH_PB_PS_PB, PH_PB_PS_PG, PH_PB_PS_PH, PH_PG_BK_PG, PH_PG_PB_BK, PH_PG_PH_GR, PH_PS_PH_PB, PH_PS_PH_PS, PN_GR_PG_PN, PN_GR_PG_WH, PN_PG_PB_PN, PN_PG_PN_GR, PN_PG_PN_HO, PN_PG_PN_PG, PN_PH_PG_PN, PS_PB_HO_PH, SC_BK_PB_BK, SC_BK_PB_GR, SC_BK_PS_SC, SC_BK_SC_BK, SC_GR_SC_GR, SC_PN_SC_PN, SC_PS_BK_SC, WH_GR_SC_PS, WH_HO_PH_PB, WH_PH_PB_HO, WH_PH_PS_PB, WH_PH_PS_SC, WH_PS_BK_SC, WH_SC_PN_WH, WH_SC_PS_SC |
| 5 | 90 | 73 | BK_SC_PS_PB_BK, GR_BK_PB_BK_PB, GR_HO_GR_PH_GR, GR_HO_PB_PG_PN, GR_HO_PH_PB_GR, GR_HO_PH_PB_PG, GR_HO_PH_PB_PH, GR_PB_BK_PB_PG, GR_PG_GR_PB_GR, GR_PG_GR_PG_GR, GR_PG_GR_PG_SC, GR_PG_PB_BK_GR, GR_PG_PB_BK_SC, GR_PG_PB_GR_SC, GR_PG_PB_PG_GR, GR_PG_PB_PG_PN, GR_PG_PB_PG_SC, GR_PG_PB_PS_BK, GR_PG_PB_PS_SC, GR_PG_PB_SC_BK, GR_PG_PH_PB_PH, GR_PG_PH_PG_PB, GR_PH_GR_SC_GR, GR_PH_PB_BK_GR, GR_PH_PB_PH_GR, GR_PH_PB_PS_SC, GR_PH_PG_PN_GR, GR_PN_BK_PB_BK, GR_PN_GR_PG_GR, GR_PN_GR_PG_PN, GR_PN_PG_PB_HO, GR_SC_PS_SC_BK, HO_GR_PG_PB_PG, HO_PB_PS_GR_BK, HO_PG_PB_PG_PB, HO_PH_GR_HO_PB, HO_PH_HO_PH_HO, HO_PH_PB_GR_PG, HO_PH_PB_PG_GR, HO_PH_PB_PG_PB, HO_PH_PB_PH_GR, HO_PH_PB_PH_HO, HO_PH_PB_PS_SC, HO_PH_PN_PG_PB, HO_PH_PS_PB_PS, PG_GR_PG_PB_PG, PG_PB_BK_SC_PB, PG_PB_BK_SC_PS, PG_PB_PG_PB_GR, PG_PB_PG_PB_PG, PG_PB_SC_BK_GR, PG_PH_PB_BK_PH, PH_GR_PG_PB_BK, PH_PB_PH_PB_PH, PH_PB_PH_PS_PG, PH_PB_PS_PB_PG, PH_PB_PS_PH_HO, PH_PB_SC_BK_SC, PH_PG_PB_PS_SC, PH_PG_PH_PB_PG, PH_PS_PB_PS_BK, PN_GR_PG_GR_PN, PN_GR_PG_PB_PG, PN_GR_PN_GR_PG, PN_GR_PN_PG_GR, PN_PG_PB_PG_PH, PN_PG_PB_PG_PN, PS_PB_BK_PS_BK, PS_SC_PS_SC_BK, SC_BK_PB_SC_BK, SC_BK_SC_BK_PS, SC_PS_SC_PS_SC, WH_PH_WH_PH_WH |
| 6 | 29 | 29 | BK_PS_BK_SC_BK_SC, GR_BK_GR_PG_BK_GR, GR_BK_PS_BK_SC_PB, GR_BK_SC_GR_BK_SC, GR_HO_GR_HO_PH_PS, GR_HO_PH_PG_PB_BK, GR_PG_GR_HO_PH_GR, GR_PG_GR_HO_PH_HO, GR_PG_GR_PG_GR_PG, GR_PG_GR_PG_HO_GR, GR_PG_GR_SC_PS_SC, GR_PG_HO_PG_PB_GR, GR_PG_PB_GR_PB_PG, GR_PG_PB_PG_PB_PN, GR_PG_PB_PS_SC_BK, GR_PG_PB_SC_BK_GR, GR_PH_PB_PS_PB_PS, GR_SC_PS_SC_BK_SC, HO_GR_PG_GR_PH_HO, HO_PH_PB_PG_GR_PN, HO_PH_PB_PH_PB_GR, HO_PH_PS_PB_PS_PH, PG_GR_PG_PB_BK_GR, PH_PB_PG_PH_PG_GR, PH_PB_PG_PS_PG_GR, PH_PG_PB_GR_PG_PB, PN_GR_PN_GR_PN_PG, PN_PG_PB_PG_PB_BK, PN_PG_PH_PB_PG_GR" |
| 7 | 12 | 12 | GR_BK_GR_BK_GR_BK_GR, GR_PG_GR_PG_GR_PG_PN, GR_PG_PB_PS_PB_PS_SC, GR_PG_PH_HO_GR_PG_GR, GR_PG_SC_PS_SC_BK_SC, HO_PH_PB_PH_PB_PB_BK, HO_PH_PB_PH_PB_PH_HO, HO_PH_PB_PH_PS_PH_PB, HO_PH_PG_PH_PG_GR_PG, HO_PN_GR_PH_PG_GR_PG, PH_PB_PH_PB_PH_PB_PG, PH_PB_PH_PB_PH_PB_PH" |
| 8 | 4 | 4 | GR_BK_WH_SC_PS_GR_WH_SC, GR_PG_PB_PG_GR_PG_PB_GR, GR_PG_PB_PG_PB_PG_PB_BK, HO_PH_GR_PG_PB_PG_PB_PG |
| 9 | 2 | 2 | BK_GR_PG_PB_GR_GR_BK_GR_BK, HO_PH_PS_PH_PS_HO_PH_PS_SC |
| 10 | 2 | 2 | GR_BK_PS_SC_BK_SC_BK_PB_BK_PB, PG_GR_PN_GR_PG_PN_PG_PB_GR_PN |
| **Total** | **4826** | **401** |  |
