# Supplementary material for: Chimpanzees produce diverse vocal sequences with ordered and recombinatorial properties
Source: Commun Biol. 2022 May 16;5:410. doi: 10.1038/s42003-022-03350-8 (PMC9110424; doi:10.1038/s42003-022-03350-8)
Supplement: Supplementary file 6 — Reporting Summary [file 42003_2022_3350_MOESM6_ESM.pdf]

nature portfolio

Corresponding author(s): Cedric Girard-Buttoz

Last updated by author(s): Mar 2, 2022

Reporting Summary

Nature Portfolio wishes to improve the reproducibility of the work that we publish. This form provides structure for consistency and transparency in reporting. For further information on Nature Portfolio policies, see our Editorial Policies and the Editorial Policy Checklist.

Statistics

For all statistical analyses, confirm that the following items are present in the figure legend, table legend, main text, or Methods section.

n/a	Confirmed	
☐	☒	The exact sample size (*n*) for each experimental group/condition, given as a discrete number and unit of measurement
☒	☐	A statement on whether measurements were taken from distinct samples or whether the same sample was measured repeatedly
☐	☒	The statistical test(s) used AND whether they are one- or two-sided<br>*Only common tests should be described solely by name; describe more complex techniques in the Methods section.*
☐	☒	A description of all covariates tested
☐	☒	A description of any assumptions or corrections, such as tests of normality and adjustment for multiple comparisons
☐	☒	A full description of the statistical parameters including central tendency (e.g. means) or other basic estimates (e.g. regression coefficient) AND variation (e.g. standard deviation) or associated estimates of uncertainty (e.g. confidence intervals)
☒	☐	For null hypothesis testing, the test statistic (e.g. *F*, *t*, *r*) with confidence intervals, effect sizes, degrees of freedom and *P* value noted<br>*Give P values as exact values whenever suitable.*
☐	☒	For Bayesian analysis, information on the choice of priors and Markov chain Monte Carlo settings
☒	☐	For hierarchical and complex designs, identification of the appropriate level for tests and full reporting of outcomes
☒	☐	Estimates of effect sizes (e.g. Cohen's *d*, Pearson's *r*), indicating how they were calculated

Our web collection on statistics for biologists contains articles on many of the points above.

Software and code

Policy information about availability of computer code

Data collection	No software were used to collect the data
Data analysis	The spectrograms from the vocal data were analyzed using PRAAT version 6.1.31. The statistical analyses were conducted using R version 3.4.4 and JASP version 0.13.1

For manuscripts utilizing custom algorithms or software that are central to the research but not yet described in published literature, software must be made available to editors and reviewers. We strongly encourage code deposition in a community repository (e.g. GitHub). See the Nature Portfolio guidelines for submitting code & software for further information.

Data

Policy information about availability of data

All manuscripts must include a data availability statement. This statement should provide the following information, where applicable:

- Accession codes, unique identifiers, or web links for publicly available datasets
- A description of any restrictions on data availability
- For clinical datasets or third party data, please ensure that the statement adheres to our policy

The datasets generated during and/or analyzed during the current study are available from the corresponding author on reasonable request.

Field-specific reporting

Please select the one below that is the best fit for your research. If you are not sure, read the appropriate sections before making your selection.

☐ Life sciences ☒ Behavioural & social sciences ☐ Ecological, evolutionary & environmental sciences

For a reference copy of the document with all sections, see nature.com/documents/nr-reporting-summary-flat.pdf

Behavioural & social sciences study design

All studies must disclose on these points even when the disclosure is negative.

Study description	We collected quantitative data on the structure of vocal sequences in wild chimpanzees.
Research sample	We sampled wild chimpanzees in Tai National Park, Ivory Coast. We chose this population since they provide the opportunity to work on three habituated groups within the same population. We studied adult chimpanzee from both sexes. The sample is representative of the studied population since we sampled every single adult with no exclusion.
Sampling strategy	The choice of animals followed as focal individuals was chosen pseudo-randomly (pending presence of individuals in the party follow on each observation day). The sample size was chosen as to maximize the number of individuals (all individuals were sampled N=46 adult chimpanzees). However, the analyses were conducted at the population level using each vocal sequence as a data point leading to a sample size of 4826 sequences. This sample size is exceptionally large for such a study and sufficient for the simple randomizations and Binomial Bayesian tests that we ran.
Data collection	All the data were collected in the wild by TB and a field assistant. They used a microphone Sennheiser ME67 connected to a Tascam DR-40X digital recorder to record the vocalizations of the chimpanzees. TB was not blind to the study hypothesis but unlike behavioral observations, vocal recordings and their structure cannot be altered by the knowledge of the observer.
Timing	The data were collected between two study periods: January-February 2019 and December 2019 to March 2020.
Data exclusions	No data were excluded from the analysis
Non-participation	The study was on wild animals so the subjects could not drop out or decline to participate. All adult individuals present in the study groups were sampled.
Randomization	Study subjects were not allocated to experimental groups

Reporting for specific materials, systems and methods

We require information from authors about some types of materials, experimental systems and methods used in many studies. Here, indicate whether each material, system or method listed is relevant to your study. If you are not sure if a list item applies to your research, read the appropriate section before selecting a response.

Materials & experimental systems

n/a	Involved in the study	
☒	☐	Antibodies
☒	☐	Eukaryotic cell lines
☒	☐	Palaeontology and archaeology
☐	☒	Animals and other organisms
☒	☐	Human research participants
☒	☐	Clinical data
☒	☐	Dual use research of concern

Methods

n/a	Involved in the study	
☒	☐	ChIP-seq
☒	☐	Flow cytometry
☒	☐	MRI-based neuroimaging

Animals and other organisms

Policy information about studies involving animals; ARRIVE guidelines recommended for reporting animal research

Laboratory animals	The study did not involve laboratory animals
Wild animals	The study was conducted on wild chimpanzees. The study was purely observational and did not involve manipulation of or interaction with the animals.
Field-collected samples	The study did not comprise field collected samples.

Ethics oversight	The ‘Ethikrat’ of the Max Planck Society approved the study.

Note that full information on the approval of the study protocol must also be provided in the manuscript.
